# Discriminative binding of tau PET tracers PI2620, MK6240 and RO948 in Alzheimer’s disease, corticobasal degeneration and progressive supranuclear palsy brains

**DOI:** 10.1038/s41380-022-01875-2

**Published:** 2022-11-29

**Authors:** Mona-Lisa Malarte, Per-Göran Gillberg, Amit Kumar, Nenad Bogdanovic, Laëtitia Lemoine, Agneta Nordberg

**Affiliations:** 1 Division of Clinical Geriatrics, Center for Alzheimer Research, Department of Neurobiology, Care Sciences and Society, Karolinska Institutet, Stockholm, Sweden; 2 Theme Inflammation and Aging, Karolinska University Hospital, Stockholm, Sweden

**Keywords:** Diagnostic markers, Neuroscience

## Abstract

Recent mechanistic and structural studies have challenged the classical tauopathy classification approach and revealed the complexity and heterogeneity of tau pathology in Alzheimer’s disease (AD) and primary tauopathies such as corticobasal degeneration (CBD) and progressive supranuclear palsy (PSP), progressing beyond distinct tau isoforms. In this multi-tau tracer study, we focused on the new second-generation tau PET tracers PI2620, MK6240 and RO948 to investigate this tau complexity in AD, CBD, and PSP brains using post-mortem radioligand binding studies and autoradiography of large and small frozen brain sections. Saturation binding studies indicated multiple binding sites for ^3^H-PI2620 in AD, CBD and PSP brains with different binding affinities (*K*_d_ ranging from 0.2 to 0.7 nM) and binding site densities (following the order: *B*_max_AD > *B*_max_CBD > *B*_max_PSP). Competitive binding studies complemented these findings, demonstrating the presence of two binding sites [super-high affinity (SHA): IC_50(1)_ = 8.1 pM; and high affinity (HA): IC_50(2)_ = 4.9 nM] in AD brains. Regional binding distribution studies showed that ^3^H-PI2620 could discriminate between AD (*n* = 6) and control cases (*n* = 9), especially in frontal cortex and temporal cortex tissue (*p* < 0.001) as well as in the hippocampal region (*p* = 0.02). ^3^H-PI2620, ^3^H-MK6240 and ^3^H-RO948 displayed similar binding behaviour in AD brains (in both homogenate competitive studies and one large frozen hemispherical brain section autoradiography studies) in terms of binding affinities, number of sites and regional patterns. Our small section autoradiography studies in the frontal cortex of CBD (*n* = 3) and PSP brains (*n* = 2) showed high specificity for ^3^H-PI2620 but not for ^3^H-MK6240 or ^3^H-RO948. Our findings clearly demonstrate different binding properties among the second-generation tau PET tracers, which may assist in further understanding of tau heterogeneity in AD versus non-AD tauopathies and suggests potential for development of pure selective 4R tau PET tracers.

## Introduction

Several neurodegenerative disorders are characterised as proteinopathies. Tauopathy is a broad term, neuropathologically classified by the type of tau isoforms present in the pathology/neurofibrillary tangles (NFTs). Tau isoforms have varying numbers of carboxy-terminal repeat domains (for example 3R or 4R) [1]. In this context, Alzheimer’s disease (AD) is associated with an abnormal accumulation of both 3R and 4R forms [2], frontotemporal dementia (FTD) has three subtypes (3R, 4R or 3R/4R) [3], and progressive supranuclear palsy (PSP) and corticobasal degeneration (CBD) are mainly characterised by the 4R form [4]. Morphologically, these isoforms constitute NFTs, whose composition can also vary at the ultrastructure level depending on the pathology: AD (paired helical filaments » straight filaments), PSP and CBD (Straight filaments » twisted filaments) [5]. The wide variability of tau protein has been clearly highlighted by recent cryo-electron microscopy (cryo-EM) studies demonstrating not only distinct tau isoforms but also distinctive folds in the tau fibrils [6, 7]. Hence, the 3R and 4R designation alone seems insufficient for discriminating among the related pathologies, and an additional hierarchical classification based on the layered organisation of the fibrils has been proposed. The tauopathies are characterised by differences in the regional spread of tau in the brain, expressed by different clinical phenotypes [8].

The recent rapid development of molecular imaging with positron emission tomography (PET) has led to in vivo PET imaging of tau in brain tissue using different tau PET tracers. The first generation of tau PET tracers, which include ^18^F-AV-1451 (flortaucipir), ^18^F-THK5317, and ^11^C-PBB3, has been followed by a second generation: ^18^F-MK6240, ^18^F-PI2620, ^18^F-RO948, ^18^F-PM-PBB3 (^18^F-APN-1607), ^18^F-GTP1, and ^18^F-JNJ31 [8]. Clarification of the clinical impact of these tau PET tracers, i.e. whether they will be able to discriminate between the tauopathies early in the course of the disease and not only at autopsy, is crucial. In vivo PET studies have indicated that ^18^F-MK6240 [9], ^18^F-PI2620 [10] and ^18^F-RO948 [11] can be used to discriminate between AD patients and healthy controls. Moreover, ^18^F-RO948 was able to detect early cognitive changes in AD patients more efficiently than magnetic resonance imaging [12]. In line with this, ^18^F-MK6240 could also detect lower tau levels, essential for early detection [13] and both ^18^F-MK6240 [14] and ^18^F-PI2620 have potential to distinguish between early and late Braak stages in AD continuum [15].

In addition, ^18^F-PI2620 PET studies have shown significantly increased binding to CBD and PSP brains compared to age-matched controls, but with a different regional pattern from that in AD brains [16, 17]. In contrast, a few PET studies have reported no binding of ^18^F-MK6240 and ^18^F-RO948 to non-AD dementia brain tissue [18]. In vitro binding studies in autopsy brain tissue can provide a deeper understanding of the properties of the second-generation tau PET tracers; several in vitro binding studies have been published, mainly using autoradiography methods often combined with AT8 immunostaining [16, 19–23]. A few in vitro binding studies in AD brain homogenates have suggested a high-affinity (HA) binding site for MK6240 (0.15-0.32 nM) [21], PI2620 (8.5 nM) [20] and RO948 (18 nM) [24]. A deeper characterisation of the binding properties of ^3^H-MK6240 in AD brain tissue revealed two binding sites for MK6240 in the hippocampus: a super-high affinity (SHA) site (1 pM; 58%) and a HA site (12 nM; 42%) [21]. There seem to be some discrepancies between in vivo and in vitro PET tracer data in the literature. In vivo PET studies with ^18^F-AV-1451 have demonstrated binding in PSP patients [25, 26] while other studies [27] reported no in vitro binding with ^18^F-AV-1451 using autoradiography in post-mortem PSP brain tissue. It was observed no in vitro binding of ^3^H-MK6240, ^3^H-PI2620 and ^3^H-RO948 using autoradiography in PSP frontal cortex (FC) [23], while binding with ^3^H-PI2620 has been reported in PSP cases [16, 22] and in CBD cases [17]. A recent comparative study performed in post-mortem human brain tissue reported that these three tau tracers bound specifically to paired helical filaments of tau, and were able to distinguish between AD and non-AD tauopathies [23], while ^18^F-AV-1451 does not show any specificity for 4R-tauopathies in post-mortem autoradiography [27].

We have also recently used in silico modelling based on known cryo-EM tau fibril structures to demonstrate that different tau PET tracers can show different preferences for and binding affinities to multiple binding sites on the tau fibrils in AD compared to CBD and PSP tissue [28–31].

This background suggests that the neuropathological differences between AD, PSP and CBD arising from the structural complexity of tau folds and isoforms remain unresolved and warrant further exploration. Early tau detection with the help of recently developed tau tracers could be a decisive diagnostic tool if the binding behaviour of these tracers is properly characterised for the different tauopathies. Hence, we decided to further explore the different binding properties/mechanisms of the second-generation tau PET tracers in AD versus in primary tauopathies such as PSP and CBD using in vitro radioligand binding in brain homogenates as well as large and small frozen brain section autoradiography, with the idea that this could also discriminate AD from other neurodegenerative diseases.

## Materials and methods

### Chemicals

^3^H-MK6240 [specific activity (SA) 45.77 Ci/mmol] was provided by Merck Sharp and Dohme Corp. (Whitehouse Station, USA). ^3^H-PI2620 (SA 43.6 Ci/mmol) was gifted by Life Science Molecular Imaging (Germany) as well as custom synthesised by Novandi (Södertälje, Sweden). ^3^H-R0948 (SA 31.0 Ci/mmol) was custom synthesised by Novandia (Södertälje, Sweden). Unlabelled MK6240 was provided by Cerveau. Unlabelled PI2620 was custom synthesised by Novandi (Södertälje, Sweden) and unlabelled R0948 was custom synthesised by F.Hoffmann-LaRoche Ltd (Basel, Switzerland). All other chemicals [tris base, disodium phosphate (Na_2_HPO_4_), potassium dihydrogen phosphate (KH_2_PO_4_), bovine serum albumin (BSA) and protease/phosphatase inhibitors] were purchased from Sigma-Aldrich AB (Sweden).

### Autopsy material

Frozen human brain tissue from eleven AD, five CBD, four PSP and eight control patients was provided by the Netherlands Brain Bank (Amsterdam, Netherlands). Frozen human brain tissue from PSP case number 26 was provided by Professor Bernadino Ghetti, the Neuropathology of Dementia Laboratory, Indiana University School of Medicine (Indianapolis, IN, USA) (see Table 1 for demographic data). Brain homogenates were prepared by homogenising the brain tissue in phosphate buffer saline (PBS; pH 7.4) containing 0.1% BSA (250 mg tissue/mL) and protease/phosphatase inhibitors (10 μL/mL); aliquots were stored at −80 °C until used in the binding experiments.

**Table 1:** Clinical demographic information.

	No	Sex (M/F)	Age (years)	ApoE (E/E)	Braak stage	Onset	Post-mortem delay (hour)
For binding studies							
AD	1	M	61	3/3	5	EOAD	*N/A*
	2	M	70	4/4	4	EOAD	4
	3	M	78	4/4	5	LOAD	7
	4	M	85	3/3	4	LOAD	*N/A*
	5	F	59	4/4	5	EOAD	4
	6	F	59	3/3	5	EOAD	11
	7	F	59	3/3	6	EOAD	4
	8	F	66	4/3	5	EOAD	7
	9	F	73	3/3	5	LOAD	2
	10	F	81	4/3	5	LOAD	6
	***Mean***	**4M/6F**	**69.1** **±** **9.8**	**5 E3/5 E4**	**4–6**	**6EOAD/4LOAD**	**6**
Large frozen sections for autoradiography							
	A	F	79	4/4	5	LOAD	16
For binding studies	11	M	62	3/3	1	*N/A*	*7*
Control	12	M	78	3/3	1	*N/A*	*<18*
	13	M	79	3/3	2	*N/A*	*9*
	14	M	81	3/3	2	*N/A*	*N/A*
	15	F	50	3/3	1	*N/A*	*4*
	16	F	71	3/3	1	*N/A*	*7*
	17	F	77	3/3	1	*N/A*	*3*
	18	F	84	3/3	1	*N/A*	*7*
	***Mean***	**4M/4F**	**72.5** **±** **11.4**	**8 E3**	**1–2**		**6**
For binding studies							
CBD	19	M	69	*N/A*	2	*N/A*	*8*
	^a^20	M	72	*N/A*	*N/A*	*N/A*	*5*
	21	M	73	*N/A*	*N/A*	*N/A*	*6*
	^a^22	F	65	*N/A*	*N/A*	*N/A*	*7*
	^a^23	F	66	*N/A*	3	*N/A*	*5*
	***Mean***	**3M/2F**	**69** **±** **3.5**		**2–3**		**6**
For binding studies							
PSP	24	M	69	*N/A*	1	*N/A*	*6*
	*25*	M	72	*N/A*	*N/A*	*N/A*	*5*
	*26*	F	64	*N/A*	*N/A*	*N/A*	*N/A*
	^a^27	F	67	*N/A*	1	*N/A*	*6*
	^a^28	F	70	*N/A*	*N/A*	*N/A*	*7*
	***Mean***	**2M/3F**	**68.4** **±** **3.0**		**1**		**6**

Bolded values show the total number of samples from male and female patients in this study (controls and patients with AD, CBD or PSP), as well as the means ± SD of age, ApoE, Braak stage, and post-mortem delay.

*AD* Alzheimer’s disease, *ApoE* apolipoprotein E, *CBD* corticobasal degeneration, *EOAD* early-onset AD, *F* female, *LOAD* late-onset AD, *M* male, *N/A* not applicable/available, *PSP* progressive supranuclear palsy.

^a^Samples also used for the small frozen section autoradiography. Sporadic Alzheimer’s disease has been also reported and described in our previous publications [21, 34, 35, 59].

Large frozen brain blocks from one 79-year-old female patient with sporadic AD were provided by the Brain Bank at Karolinska Institutet (Stockholm, Sweden). The AD patient had been clinically followed by AN at the Memory clinic, Karolinska University Hospital, (Stockholm, Sweden) and the clinical and pathological data have been described previously [32] (Table 1).

### Brain sectioning

Large frozen brain blocks were dipped into Carboxymethyl cellulose gel and sectioned as decribed in Gillberg et al, 1986 [33]. Hemispherical brain sections (100 μm thick) were cut using a cryomacrotome (Leica CM3600 XP). A thin paper on an adhesive tape was placed on the large frozen hemispherical brain section and the latter was transferred as quickly as possible onto a glass plate.

Small frozen sections (20 μm thick) were cut using a cryostat (Microm HM500M, Cellab) from a small fresh tissue block of frontal cortex.

### Saturation binding assays

Saturation binding assays were performed in two AD, two PSP and two CBD FC brain homogenates (0.5 mg tissue) using a ^3^H-PI2620 concentration range of 0.05 nM to 10 nM and incubation for 2 hours at room temperature (RT) with PBS and 0.1% BSA. Non-specific (NSP) binding was determined using 1μM of unlabelled PI2620. The assay was terminated as described previously [21, 32, 34, 35]. ^3^H-PI2620 binding was measured one day later in a scintillation counter (PerkinElmer Tri-Carb 2910TR). The dissociation constant (*K*_d_) and maximum number of binding sites (B_max_) were analysed using GraphPad Prism 9 software and a non-linear regression model. The Scatchard plots were prepared using GraphPad Prism 9 software. For AD and CBD data, the second binding site was drawn manually considering all the data points, including one point from the initial linear curve of the Scatchard plots.

### Competitive binding assays

Competitive binding assays on four AD FC brain homogenates [two early-onset (EO) AD and two late-onset (LO) AD; 0.5 mg tissue] were performed using 0.6 nM of ^3^H-PI2620 and increasing concentrations (1 × 10^−18^ M to 1 × 10^−6^ M) of unlabelled PI2620, unlabelled MK6240 and unlabelled RO948 for 2 h at RT. The binding assay was accomplished as described above. The half-maximal inhibitory concentration (IC_50_) was measured using the 2-site competition model in GraphPad Prism 9 software.

Competitive binding assays were also carried out under the same conditions, on three PSP FC homogenates and three CBD FC homogenates using 0.6 nM and 1 nM of ^3^H-PI2620, respectively, in the presence of increasing concentrations of unlabelled PI2620 (1 × 10^−18^ M to 1 × 10^−6^ M). The 2-site competition model in GraphPad Prism 9 software was used to plot the AD, CBD, and PSP data.

### ^3^H-PI2620 brain regional binding studies

Regional distribution studies with ^3^H-PI2620 (0.6 nM) were performed on homogenates from different regions of AD and control brains [FC (eight AD; nine controls); temporal cortex (TC) (nine AD; nine controls); parietal cortex (PC) (six AD; nine controls); hippocampus (seven AD; nine controls); and cerebellum (six AD; six controls)] in the presence of 1μM unlabelled PI2620 to determine NSP binding. The final steps of the experiment were carried out as described above. The AD group was then divided in Early-Onset of AD (EOAD), Late-Onset of AD (LOAD) groups. Each data point representing one case (three experiments in triplicate for each case). The data were analysed using GraphPad Prism 9 software.

### Large frozen adjacent hemispherical brain section autoradiography

Large frozen adjacent hemispherical coronal brain sections from AD case A (100 μm thick) were exposed to ^3^H-MK6240 (1 nM), ^3^H-PI2620 (0.3 nM) or ^3^H-RO948 (1 nM) in PBS, 0.1% BSA and 2% DMSO buffer for 1 hour at RT. The NSP binding was determined with 1μM of unlabelled MK6240, unlabelled PI2620 or unlabelled RO948, respectively. The experiments were carried out as described in previous studies [21, 32, 34, 35]. The plates were scanned with a BAS-2500 phospho-imager and the images were analysed using multigauge software. For semi-quantitative analyses, the regions of interest (ROIs) were drawn manually using multigauge software. The phosphostimulated luminescence (PSL)/mm^2^ data were transformed into fmol/mg, considering the SA of each tracer.

### Small PSP and CBD section autoradiography

Autoradiography on small frozen FC sections (20 μm thick) from three CBD and two PSP cases, using 1 nM of ^3^H-PI2620, ^3^H-MK6240 or ^3^H-RO948, was carried out using the same protocol as above. The NSP binding was determined with 1μM of unlabelled PI2620, unlabelled MK6240 or unlabelled RO948, respectively.

### Immunostaining on small frozen sections

Immunostaining was performed on adjacent frozen CBD and PSP brain sections, as used for the autoradiography studies. The sections were peroxidase-blocked for 5 min and protein-blocked using 5% non-fat dried milk powder at RT. The sections were exposed overnight at 4 °C to primary antibodies raised in mouse to target 3R + 4R tau (AT8; 1:1000 Cat.#MN1020, Invitrogen). Exposure to secondary antibody envision mouse HRP (Cat.#K4001 DAKO, Agilent Technologies) lasted 30 min at RT. The reaction was visualised by developing the sections in Peroxidase-blocking solution (Dako REAL). Between the different steps the sections were thoroughly washed in Tris Buffered Saline 1X + 0.05% Tween20 (TBST). Finally, the sections were dehydrated and mounted in cytosealXYL. All sections were treated simultaneously under the same conditions.

### Statistical analysis

The regional binding differences for ^3^H-PI2620 in EOAD, LOAD and control patients were statistically analysed using two-way ANOVA (with Sidak´s multiple comparison test) analysis in GraphPad Prism 9 software. An ANOVA *p* < 0.05 was considered significant. The data are presented as means of three experiments in triplicate with scatter dot plots.

## Results

### ^3^H-PI2620 saturation studies in AD, CBD and PSP brains

**Fig. 1:**
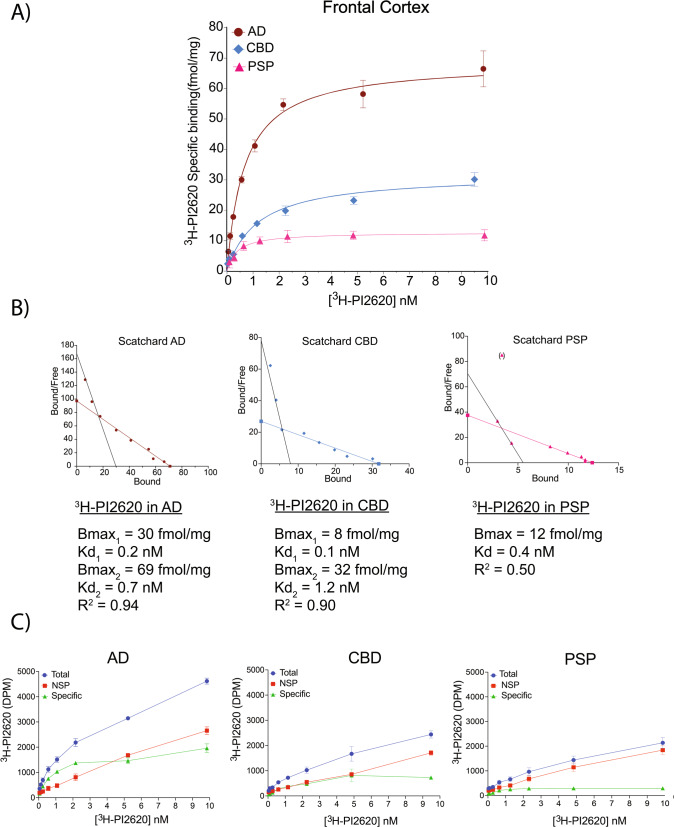
^3^H-PI2620 saturation binding assays in AD, CBD and PSP brain homogenates. **A** ^3^H-PI2620 saturation binding assays were carried out in frontal cortex brain tissue homogenates from two AD patients (5; A), two PSP patients (25; 26) and two CBD patients (20; 23) using concentrations of 0.05 to 10 nM. NSP binding was determined using 1 μM of unlabelled PI2620. **B** Scatchard plots indicating *B*_max_ and *K*_d_ values. Error bars represent the means ± SEM for three experiments in duplicate and triplicate. **C** Graphs showing total, NSP and specific binding of ^3^H-PI2620 in homogenates from two AD patients (5; A), two CBD patients (20; 23) and two PSP patients (25; 26) using concentrations of 0.05 to 10 nM. AD Alzheimer’s disease, *B*_max_ maximum number of binding sites, CBD corticobasal degeneration, DPM disintegration per minute, *K*_d_ dissociation constant, NSP non-specific, PSP progressive nuclear palsy, *R*^2^ regression coefficient, SEM standard error of the mean.

The saturation binding study results for ^3^H-PI2620 in AD, PSP and CBD FC brain homogenates and the corresponding Scatchard plots are shown in Fig. 1A and Fig. 1B, respectively. For the AD tissue, saturation occurred at a *K*_d2_ of 0.7 nM and *B*_max2_ of 69 fmol/mg. A second binding site was observed when the Scatchard plot was manually drawn (*K*_d1_ = 0.2 nM; and *B*_max1_ = 30 fmol/mg). For the CBD tissue, saturation occurred at a *K*_d2_ of 1.2 nM and *B*_max2_ of 32 fmol/mg. The Scatchard plot revealed a second binding site (*K*_d1_ = 0.1 nM; and *B*_max1_ = 8.0 fmol/mg). For PSP tissue, the saturation curve displayed one binding site with a *K*_d_ of 0.3 nM and *B*_max_ of 12 fmol/mg. The total, NSP and specific binding results for ^3^H-PI2620 are shown in Fig. 1C. For the AD tissue, total binding increased with increasing ^3^H-PI2620 concentrations and NSP binding increased linearly, while specific binding increased up to a concentration of 2 nM where it reached a plateau. The total binding for the CBD tissue was similar to that for the PSP tissue, but NSP binding was much higher in PSP tissue than in CBD tissue.

### ^3^H-PI2620 comparative competitive studies with unlabelled MK6240 and RO948 in AD brains

**Fig. 2:**
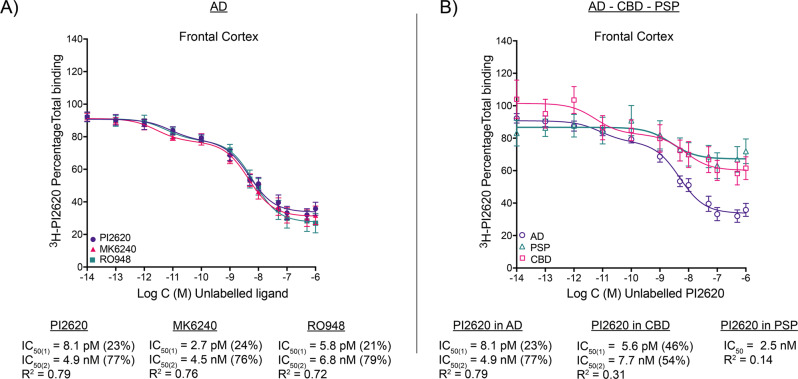
^3^H-PI2620 competitive binding assays with unlabelled tau PET tracers PI2620, MK6240, RO948. **A** ^3^H-PI2620 competitive binding assays in frontal cortex brain homogenates from four AD patients (1; 3; 5; A) using a single concentration of ^3^H-PI2620 (0.6 nM) and increasing concentrations of unlabelled PI2620, MK6240 and RO948 (1 × 10^−14^ M to 1 × 10^−5^ M). **B** ^3^H-PI2620 competitive binding studies in frontal cortex brain homogenates from three PSP patients (26; 25; 28) and three CBD patients (19; 20; 23) using a single concentration of ^3^H-PI2620 (0.6 nM for PSP; 1 nM for CBD) and increasing concentrations of unlabelled PI2620. The fraction of high affinity sites is expressed as a percentage for each unlabelled compound. Error bars represent the means ± SEM from three experiments in triplicate for each unlabelled compound. AD Alzheimer´s disease, CBD corticobasal degeneration, IC_50_ half-maximal inhibitory concentration, PSP progressive supranuclear palsy, *R*^*2*^ regression coefficient, SEM standard error of the mean.

We further analysed the binding behaviour of ^3^H-PI2620 in AD brains by comparing its binding properties to those of MK6240 and RO948 in the same AD tissue. Competitive binding studies were carried out in the FC with ^3^H-PI2620, using increasing concentrations of unlabelled MK6240 or RO948 in the same tissue. The results are shown in Fig. 2A. MK6240 competed with ^3^H-PI2620 for two binding sites (IC_50(1)_ = 2.7 pM; and IC_50(2)_ = 4.5 nM) and RO948 competed with ^3^H-PI2620 for two binding sites (IC_50(1)_ = 5.8 pM; and IC_50(2)_ = 6.8 nM), compared with unlabelled PI2620 versus ^3^H-PI2620 (IC_50(1)_ = 8.1 pM; and IC_50(2)_ = 4.9 nM). Thus, unlabelled MK6240, RO948 and PI2620 demonstrated comparable binding affinities for two binding sites with similar proportions binding to the SHA (>20%) and HA (>70%) binding sites.

### ^3^H-PI2620 competitive binding studies in AD, CBD and PSP brain tissue

To further characterise the binding properties of ^3^H-PI2620 in PSP and CBD compared to AD FC brain tissue, competitive studies using increasing concentrations of unlabelled PI2620 versus ^3^H-PI2620 were carried out in CBD and PSP brain tissue. The results are shown in Fig. 2B. In CBD tissue, PI2620 competed in affinity for two binding sites (IC_50(1)_ = 5.6 pM; and IC_50(2)_ = 7.7 nM). These were in the same range as those reported above for AD tissue (IC_50(1)_ = 8.1 pM; and IC_50(2)_ = 4.9 nM). In PSP tissue, unlabelled PI2620 competed for only one binding site, in the nanomolar range (IC_50_ = 2.5 nM), which was comparable to the HA IC_50(2)_ for CBD and AD in FC tissue. When the proportions of SHA and HA binding sites were calculated, AD FC tissue expressed 23 % SHA and 77 % HA, while CBD expressed 46% SHA and 54 % HA sites. For PSP only one HA site was detected.

### ^3^H-PI2620, ^3^H-MK6240 and ^3^H-RO948 autoradiography on large frozen adjacent hemispherical brain sections from a patient with sporadic AD

**Fig. 3:**
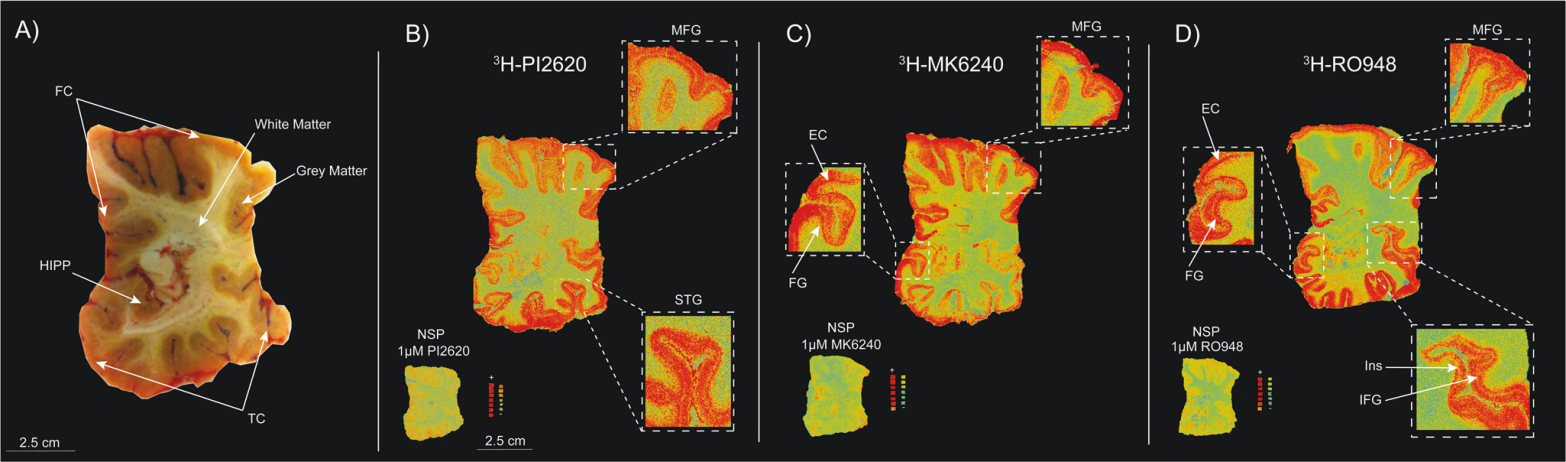
Comparative ^3^H-PI2620, ^3^H-MK6240 and ^3^H-RO948 autoradiography studies on large frozen adjacent brain sections from a sporadic AD patient. **A** Frozen hemispherical brain block dipped into carboxyl methyl cellulose gel prior to sectioning showing the cortical regions and the border between the grey and white matter. Autoradiography was performed on large frozen adjacent hemispherical brain sections from AD case A using **B** ^3^H-PI2620 (0.3 nM), **C** ^3^H-MK6240 (1 nM), and **D** ^3^H-RO948 (1 nM); 1 μM of unlabelled PI2620, MK6240 and RO948, respectively, was used to visualise the NSP binding. As the concentration used for each tracer was different, autoradiography images were not set on the same colour/brightness threshold levels for ^3^H-PI2620 (standards: + = 6119.9 fmol/mg, ‒ = −1.6 fmol/mg), ^3^H-MK6240 (standards: + = 6870.7 fmol/mg, ‒ = −4.5 fmol/mg) and ^3^H-RO948 (standards: + = 8233.8 fmol/mg, ‒ = −2.7 fmol/mg). ^*3*^*H-MK6240 autoradiography was adapted from* [21]. AD Alzheimer’s disease, EC entorhinal cortex, FC frontal cortex, FG fusiform gyrus/lateral occipitotemporal gyrus, HIPP hippocampus, IFG inferior frontal gyrus, Ins insula, MFG middle frontal gyrus, NSP non-specific binding [red (+) = highest binding; blue (‒) = lowest binding], STG superior temporal gyrus, TC temporal cortex.

^3^H-PI2620, ^3^H-MK6240 and ^3^H-RO948 autoradiography results from large frozen adjacent hemispherical brain sections from a 79-year-old female patient with sporadic LOAD (AD case A; Table 1, Fig. 3A) are shown in Fig. 3. Initial visual/qualitative assessment of the total binding of ^3^H-PI2620 (Fig. 3B**)**, ^3^H-MK6240 (Fig. 3C**)**, and ^3^H-RO948 (Fig. 3D) revealed similar binding patterns for all three tau tracers in the frontal and temporal lobes with a clear laminar binding pattern for all three tracers. The highest binding intensity was found in the deeper (5 and 6) and superficial (1, 2 and 3) cortical layers while there was a lower binding in layer 4 of the cortex. For all three tracers, the binding became more intense in the superficial layers of the associative regions of the neocortex. The cortical NSP binding (visually) was low for all three tracers.

### ^3^H-PI2620, ^3^H-MK6240 and ^3^H-RO948 autoradiography binding comparison in different layers of distinct cortical regions on large frozen adjacent hemispherical brain sections from a patient with sporadic AD

**Fig. 4:**
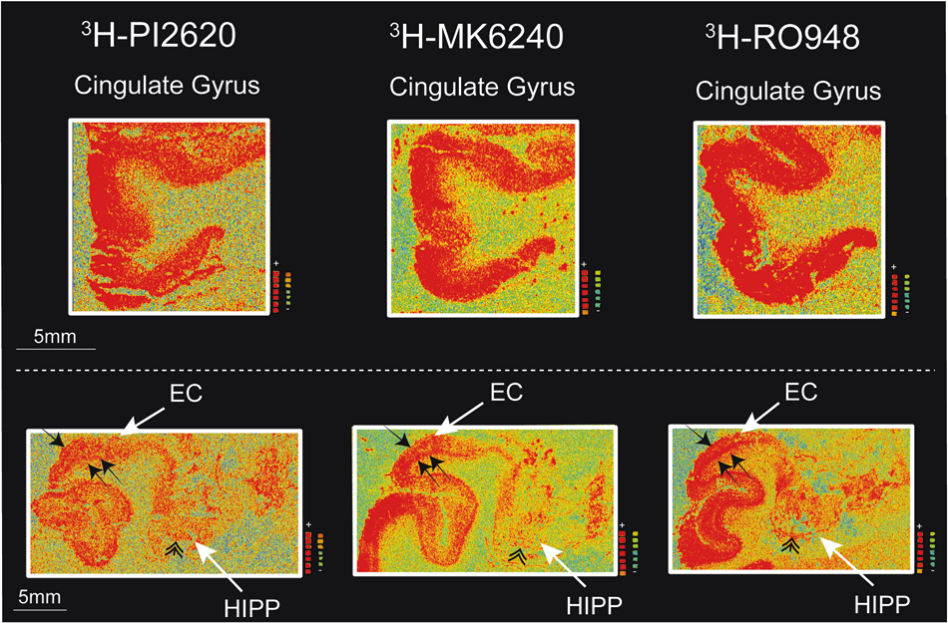
^3^H-PI2620, ^3^H-MK6240 and ^3^H-RO948 autoradiography binding comparison in different layers of distinct cortical regions of large frozen adjacent hemispherical brain sections from a patient with sporadic AD. Autoradiography images are extracted from Fig. 3. Only the total binding for the three tracers in different regions of interest (ROI) is shown. The white arrows indicate the ROI. The *small black single arrow* indicates the superficial layers of the cortex, the *small black double arrows* indicate the deep layers of the cortex and the *double arrowhead* indicate the hippocampal CA1 region. AD Alzheimer’s disease, EC entorhinal cortex, FC frontal cortex, FG fusiform gyrus/lateral occipitotemporal gyrus, HIPP hippocampus.

In cingulate gyrus, where the layer 4 of the cortex is absent, all three tau tracers bound strongly throughout all layers (Fig. 4; *top panel*). Similar qualitative/visual assessment in the entorhinal cortex (EC), showed high binding of ^3^H-MK6240 and ^3^H-RO948 in all layers; with a specifically stronger binding in the deep layers (Fig.4; *lower panel, small double arrows*). The ^3^H-PI2620 binding was less intense and more diffuse in all EC layers (Fig. 4; *lower panel*). Moreover, using ^3^H-PI2620, the discrimination between binding in superficial (*small single arrow*) and deep layers (*double single arrow*s) was very subtle/non-existent compared to ^3^H-MK6240 and ^3^H-RO948 binding, where a clear distinction was observed between superficial and deep layers (Fig. 4; *lower panel*). Similarly, in the hippocampal (HIPP) CA1 region *(double arrowhead)*, ^3^H-PI2620 demonstrated qualitatively less binding than ^3^H-MK6240 and ^3^H-RO948 (Fig. 4; *lower panel*).

### ^3^H-PI2620 regional distribution in EOAD, LOAD and control brains

**Fig. 5:**
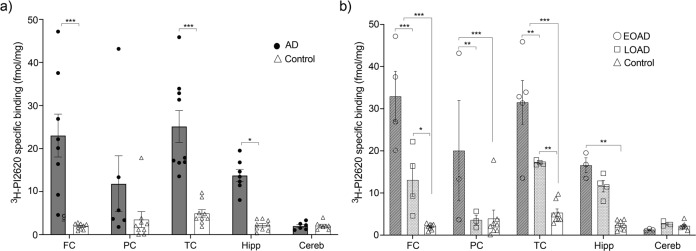
^3^H-PI2620 regional distribution binding assays in brain homogenates of EOAD, LOAD and controls. ^3^H-PI2620 regional distribution binding studies in post-mortem frontal cortex, parietal cortex, temporal cortex, hippocampus and cerebellum brain homogenates from AD patients and controls were carried out using a single concentration of ^3^H-PI2620 (0.6 nM) and unlabelled PI2620 (1 *μ*M to determine NSP binding). **a** The comparison of ^3^H-PI2620 specific binding (fmol/mg) in brain homogenates from AD (dark grey) and control cases (no shaded). **b** AD group divided in EOAD (dark shaded), LOAD (light shaded) and control (no shaded) cases. The data are presented as scatter dot plots with error bars representing the means ± SEM from 3 experiments in triplicate for each case. ***p < 0.001, **p < 0.01, *p = 0.02. AD Alzheimer’s disease, Cereb cerebellum (3 EOAD, 3 LOAD, 7 controls), EOAD early-onset AD, FC frontal cortex (4 EOAD, 4 LOAD, 8 controls), Hipp hippocampus (3 EOAD, 4 LOAD, 8 controls), LOAD late-onset AD, PC parietal cortex (3 EOAD, 3 LOAD, 8 controls), TC temporal cortex (5 EOAD, 4 LOAD, 8 controls).

The regional binding of ^3^H-PI2620 was analysed in five regions (FC, PC, TC, hippocampus and cerebellum) of brains from AD patients with EOAD or LOAD and controls. The results are presented in Fig. 5a, b in the FC and TC. ^3^H-PI2620 showed significantly higher specific binding in FC and TC (*p* < 0.001) as well as in hippocampus (*p* = 0.02) of AD brains compared to control brains (Fig. 5a**)**.^3^H-PI2620 showed significantly higher specific binding in the FC (*p* < 0.001), PC and TC (*p* < 0.01) of EOAD brains than in LOAD brains. ^3^H-PI2620 binding was significantly higher in all cortical regions (*p* < 0.001) and in the hippocampus (*p* < 0.01) of EOAD brains than in control brains. Binding was significantly higher in LOAD brains than in controls in the FC (*p* = 0.02) and TC (*p* < 0.01) (Fig. 5b**)**. No significant differences in ^3^H-PI2620 binding in the cerebellum were observed between AD and control brains.

### ^3^H-PI2620, ^3^H-MK6240 and ^3^H-RO948 comparative autoradiography in CBD and PSP brains

**Fig. 6:**
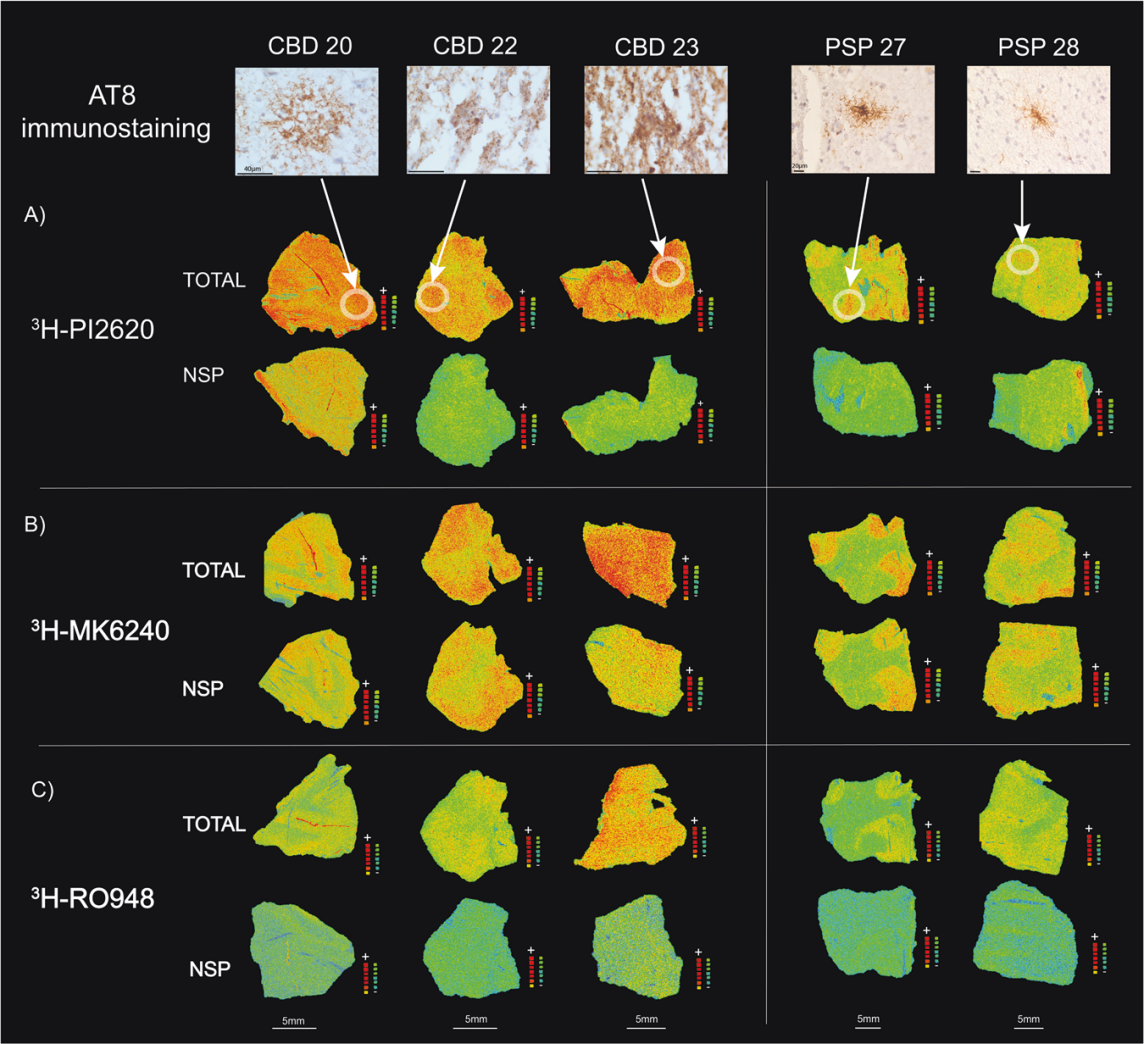
Comparative ^3^H-PI2620, ^3^H-MK6240 and ^3^H-RO948 autoradiography and corresponding AT8 staining on small frozen post-mortem frontal brain sections from CBD and PSP patients. Autoradiography was carried out on small frozen adjacent frontal cortex sections from CBD and PSP patients using **A** ^3^H-PI2620 (1 nM); **B** ^3^H-MK6240 (1 nM); and **C** ^3^H-RO948 plus 1 μM of unlabelled PI2620, MK6240 and RO948, respectively. Autoradiography colour/brightness threshold levels were set the same for the three tracers: ^3^H-PI2620 standards: + = 5898.5 fmol/mg, ‒ = −5.7 fmol/mg; ^3^H-MK6240 standards: + = 5555.9 fmol/mg, ‒ = −3.1 fmol/mg; and ^3^H-RO948 standards: + = 8181.5 fmol/mg, − = −0.3 fmol/mg. For each tracer, the upper row represents total binding, and the lower row represents NSP binding. AT8 (pS202/pT205) staining (1:1000) performed on adjacent frozen frontal cortex sections from CBD patients 20, 22, and 23, and PSP patients 27 and 28 are presented as pathology references (magnification ×40 for all images). The same scale was used for all CBD (40*μ*m) and PSP (20 μm) samples. CBD corticobasal degeneration, NSP non-specific, PSP progressive supranuclear palsy.

The ^3^H-PI2620, ^3^H-MK6240 and ^3^H-RO948 autoradiography results from small frozen FC sections from the brains of three CBD and two PSP patients are shown in Fig. 6. Visual assessment of the results for the CBD and PSP tissue demonstrated that ^3^H-PI2620 binding was significantly more intense for total than for NSP binding. For ^3^H-RO948, some differences were observed between total and NSP binding but to a much lower extent than for ^3^H-PI2620. For ^3^H-MK6240, both total and NSP binding intensities were high in most cases. A quantitative evaluation of the binding properties of ^3^H-MK6240 and ^3^H-PI2620 was performed; total, NSP and specific binding values in fmol/mg are shown in Table 2. The percentage of specific binding for ^3^H-PI2620 (Fig. 6A) was very high in CBD samples (CBD 20: 58%, CBD 22: 77% and CBD 23: 71%). The percentage of specific binding for ^3^H-MK6240 (Fig. 6B) was lower (CBD 20: 24%, CBD 22: 0% and CBD 23:29%). The findings were similar in PSP tissue; there was a high percentage of specific binding for ^3^H-PI2620 (PSP 27: 73%, PSP 28: 44%) and a lower percentage of specific binding for ^3^H-MK6240 (PSP 27: 18%, PSP 28: 12%). The specific binding for ^3^H-PI2620 in the CBD samples ranged from 27.6 to 62.3 fmol/mg and 20.3 to 34.9 fmol/mg in PSP samples while, for ^3^H-MK6240, the specific binding was lower, ranging from 0 to 19.9 fmol/mg in CBD samples and from 4.6 to 6.5 fmol/mg in PSP samples.

**Table 2:** Comparative ^3^H-PI2620 and ^3^H-MK6240 binding studies expressed as total (fmol/mg), non-specific (NSP; fmol/mg) and specific (fmol/mg and %) binding in small frozen sections from CBD and PSP brains.

Diagnostic group	TOTAL (fmol/mg)		NSP (fmol/mg)		Specific (fmol/mg)		Specific (%)	
	^3^H-PI2620	^3^H-MK6240	^3^H-PI2620	^3^H-MK6240	^3^H-PI2620	^3^H-MK6240	^3^H-PI2620	^3^H-MK6240
CBD 20	47.6	39.1	19.9	29.9	27.6	9.2	58.1	23.6
CBD 22	71.3	44.5	16.5	51.3	54.9	0.0	76.9	0.0
CBD 23	87.6	69.7	25.3	49.8	62.3	19.9	71.1	28.6
PSP 27	47.2	35.6	12.3	29.1	34.9	6.5	73.9	18.4
PSP 28	46.3	39.1	26.0	34.5	20.3	4.6	43.9	11.8

*CBD* corticobasal degeneration, *PSP* progressive supranuclear palsy.

### Tau immunohistochemistry in CBD and PSP brains

AT8 staining results for each case are shown in Fig. 6. The specific positivity of the AT8 immunostaining confirmed the diagnosis of PSP and CBD, by visualisation of disease specific tau changes, tufted astrocytes and astrocytic plaques considered as the morphological hallmarks of PSP and CBD respectively.

High AT8 staining was observed in the FCs, predominantly throughout the cortex of the three CBD cases [20, 22, 23], with specific staining of astrocytic plaques (see Fig. 6; Supplementary Fig.1) and some neurones (data not shown). Moreover, we also observed numerous AT8 positive neuropil threads. The FCs from the two PSP brains [27, 28] showed more AT8 specific binding in the cortex than in the white matter; and mostly located in astrocyte-like structures: tufted astrocytes (see Fig. 6; Supplementary Fig. 1).

## Discussion

Recent cryo-EM studies have clearly highlighted the complexity of tau protein and demonstrated how tau folds/conformations can vary among the different tauopathies and across different brain regions [7, 36]. The main impact of this complexity can be seen in clinical settings where confounding factors between tauopathies often lead to a misdiagnosis [37, 38]. Corticobasal syndrome (CBS), whose main clinical features are a progressive unilateral or asymmetrical rigidity and apraxia [39, 40], can appear in different subtypes of neurodegenerative disease [40]. For example, amongst patients clinically diagnosed with CBS, only 50% will have a confirmed CBD diagnosis [41]. In this regard, PET imaging is a promising asset which will not only assist in early tau detection but could also help differentiate between tauopathies, with specific tracers (3R, 4R and 3R + 4R) targeting different tau isoforms [42]. Most tau tracers are still limited to clinical experimental PET studies, except flortaucipir which was approved by the FDA in 2020 for clinical practice as a PET tracer for tau in memory assessments. In this study, we aimed to perform in-depth evaluations of three second-generation tau PET tracers (PI2620, MK6240 and RO948) in AD, CBD and PSP brain tissue using in vitro imaging and immunohistochemistry techniques.

^3^H-PI2620 saturation binding studies in the frontal cortex of AD brains demonstrated the presence of two binding sites and competition binding studies with unlabelled PI2620 versus ^3^H-PI2620 complemented the saturation data, demonstrating the presence of two binding sites (SHA and HA) in AD brains, with more than 70% corresponding to the HA site. These findings are in accordance with our earlier in silico data reports, suggesting that different tau tracers can detect multiple binding sites of AD tau fibril [28–31]. Moreover, tau spreads throughout the brain in the late stages of AD, with a high number of intraneuronal tau inclusions and extracellular ghost tangles made of 3 R and 4R isoforms in the cortical and hippocampal regions [43]. In addition, the severity of cognitive impairment in AD is reasonably well correlated with in vivo tau PET imaging [44, 45] as well as Braak stages [46–48].

We also compared the binding properties of ^3^H-PI2620 with those of other 3R/4R tau tracers in the FC of AD brains, as in our earlier studies with MK6240 in the same AD patients’ brains [21]. Unlabelled RO948 competed for two binding sites with analogous affinities and, as before, our competitive studies between ^3^H-PI2620 and unlabelled MK6240 revealed two binding sites with affinities similar to those of unlabelled PI2620. Interestingly, in addition to the similar affinities for the three tau tracers in AD brains, the proportions of SHA and HA binding sites were also similar for the three tracers (the HA site accounted for more than 70% of the sites), stressing their similar binding behaviour in AD brains, rich in 3R and 4R isoforms. In our previous study, we showed that ^3^H-MK6240 had equivalent affinity (*K*_d_ = 0.3 nM) in the TC of AD brains [21]. Autoradiography of large frozen adjacent hemispherical brain sections from a sporadic AD case corroborated these results. ^3^H-PI2620, ^3^H-MK6240 and ^3^H-RO948 demonstrated the same binding pattern (visually/qualitatively) in the frontal and temporal lobes (neocortex) but a different binding pattern in the hippocampus and entorhinal cortex (allocortex). The three PET tracers all showed similar laminar patterns in the frontal and temporal lobes, with a higher binding intensity in the deeper cortical layers. Similar laminar distribution binding pattern was observed with the tau PET tracer THK5117 [35].

Interestingly, hippocampal CA1 region and entorhinal cortex displayed less ^3^H-PI2620 binding than ^3^H-MK6240 and ^3^H-RO948 despite the fact that tau tangles should dominate in these regions. The distinct morphological features of very advanced tau pathology in the hippocampus CA1 and entorhinal cortex [49] might explain the differences in binding properties between the tracers in these regions.

Our competitive and autoradiography studies in AD brains clearly demonstrated that ^3^H-PI2620, ^3^H-MK6240 and ^3^H-RO948 behaved in a similar fashion. However, earlier in silico studies suggested that, despite their comparable affinities, binding sites (number and %) and patterns, these binding sites could be different, with different binding mechanisms. In silico modelling of four HA binding sites on the AD tau fibril suggested that MK6240 binds preferentially to site 1, while RO948 (former name 6958948) binds to site 3 [30].

Moreover, competition studies with different unlabelled tau tracers (MK6240 versus PI2620 for example) can generate different proportions of subpopulations of binding sites depending on which radiotracer (^3^H-MK6240 versus ^3^H-PI2620) is used in the competition studies. Although the binding sites seem to be similar for MK6240 and PI2620, they are not exactly the same (as also observed in published in silico studies; see Zhou et al. 2021 [31]). Thus, competition studies with the same unlabelled compound as the tritiated generate different binding properties that in competition studies with a different labelled compound. Our ^3^H-PI2620 regional distribution studies in the five regions of interest (frontal, temporal and parietal cortices, hippocampus and cerebellum) demonstrated that AD and control cases can be differentiated by ^3^H-PI2620, especially in the FC and TC regions (*p* < 0.001) and the hippocampal region (*p* = 0.02). This observation is in accordance with in vivo PET studies in AD and control patients [50, 51]. Interestingly, when we divided the AD cases according to disease onset (EOAD before and LOAD after 65 years of age), binding of ^3^H-PI2620 was significantly higher in EOAD brains than in LOAD brains in the cortical regions (FC: *p* < 0.01, PC and TC: *p* < 0.01) but not in the hippocampus. The lower binding in the hippocampus could be, firstly, due to use of post-mortem brain tissue, where we target tau fibrils at the end stages of the disease and hippocampus being primarily affected regions may have been severely atrophied. Secondly, as we have discussed above, it is possible that these tracers might not target or can access all types of binding sites [28, 31, 52] on distinct tau tangles depending upon their morphology/conformation and maturity stages (ranging from pre-tangles to ghost tangles [53]). The ^3^H-PI2620 regional binding distribution followed the rank order: FC > TC > PC > hippocampus, although there was significant case-by-case variability which could be attributed to AD tau heterogeneity (with regards to folds/strains/isoforms) in different regions of the brain. A recent study by Kim et al. [36] showed that this variability could be the result of diverse structural strains of tau protein, which could dictate the rate of AD progression. They found that distinct misfolded populations of 4R-rich isomers were more prominent in rapidly pathology-driven disease. In the context of our saturation and competitive binding studies, which showed high specificity of the tracer for pathological tau, there was no significant (or potential off-target) binding in AD or control cerebellum tissue. In our previous study [21], ^3^H-MK6240 also displayed significantly higher binding in the cortical regions of EOAD brains, complementing the above findings that the binding behaviour and discriminative properties of ^3^H-PI2620 and ^3^H-MK6240 are comparable in AD. In vivo PET studies have also shown higher tau PET tracer binding in EOAD tissue than in LOAD tissue [54, 55]. The mechanistic studies of second-generation tau tracers in primary tauopathies are still evolving and some disagreement can be observed between recently published in vivo and in vitro studies, as mentioned in the introduction [16, 17, 22, 25–27]. This motivated us to investigate these tracers in primary tauopathies such as PSP and CBD.

^3^H-PI2620 saturation binding studies in the FC of CBD brains demonstrated the presence of two binding sites, as in AD brains but only one site in PSP brains. The binding affinities of the binding sites in CBD and PSP brains were almost comparable to those in AD brains (*K*_d_ ranging from 0.1 to 1.2 nM). The main difference was in the *B*_max_ (site density) values. The *B*_max_ in CBD brain tissue was 2-fold lower than that in AD brain tissue whereas, in PSP, *B*_max_ was almost 6-fold lower than in AD brain tissue. Thus, *B*_max_ was 2.6-fold higher in CBD than in PSP brain tissue. These observations were confirmed by comparing the specific and NSP binding of ^3^H-PI2620 in these cases. In AD brain tissue, the specific binding was 2.5-fold and 6.5-fold higher than in CBD and PSP brain tissues, respectively.

CBD brains commonly present with high tau levels in the glial cells and neurons of the FC as well as in the basal ganglia and brainstem. However, typical PSP brains primarily present with neurofibrillary tangles in the neurons of the basal ganglia, diencephalon and brainstem [56], which could explain the lower numbers of ^3^H-PI2620 binding sites in our saturation studies in the FC of PSP brains. FC was chosen for saturation studies since it was affected in all three pathologies albeit differently. Nevertheless, although the number/density of binding sites might vary by brain region, ^3^H-PI2620 bound to tissue from the three pathologies with similar high affinity, suggesting the presence of either specific or completely different tau-binding sites (with the same affinity) in these diseases.

Competitive studies with unlabelled PI2620 in CBD brain tissue also showed two binding sites, revealing a SHA site in the picomolar range that was not detectable in our saturation studies. In PSP, only one HA site was observed. Despite the differences in the binding affinity (for two binding sites) of PI2620 in CBD brain tissue, the proportions of the sites were equivalent. In silico studies on CBD tau fibrils predicted that, theoretically, among six potential binding sites, only two sites would be favourable for PI2620 (one entry site and one surface site) [31], while in AD tau fibrils where 12 binding sites would be available, PI2620 would preferentially bind to the core site and the entry site [28]. These findings clearly highlight the importance of the accessibility of tau-binding sites and fold conformational variability in these pathologies. In AD pathology, ghost tangles are present in heavily affected regions such as hippocampal CA1 and entorhinal cortex when the neurones have died but can hardly be visualised by AT8 staining since the epitopes of interest are washed out by proteases [49]. In this study we clearly observed in AD autoradiography that ^3^H-PI2620 binding differed between the different cortical areas, highlighting the possibility for this tracer to differentially bind to distinct tau formations.

Although the PET tracer observations from our binding and autoradiography studies complemented each other well, we observed quite low specific binding values (in saturation and regional distribution binding assays) for ^3^H-PI2620 in brains from pathologically confirmed AD, CBD and PSP cases. The specific binding values were <70 fmol/mg, <30 fmol/mg, and <10 fmol/mg for AD, CBD and PSP brains, respectively, which may be somewhat surprising considering the high Braak stages (4-6; Table 1) and the higher binding values of the tracers in small section autoradiography studies (Fig. 6).

The binding assays on brain homogenates were sensitive enough to provide important information regarding the binding characteristics (*B*_max_ and *K*_d_) of the tracers. However, we believe that the low binding could be the outcome of various experimental factors. (1) The process of preparing the brain homogenates may have caused tissue rupture as a result of sheer force and solvent exposure which, despite our taking countermeasures, could have affected the protein conformations and folds (please refer to Timasheff et al. for further clarification [57]), thereby affecting the accessibility of the tracer to the binding sites. (2). The amount of tissue used for these experiments was quite low (0.5 mg), despite having been decided on by rigorous protocol standardisation and test experiments. As we have seen in several of our previous studies, increasing the amount of tissue resulted in more NSP binding of the tracers. (3) There may also have been interference from other regions (low in target) that were part of the ROI. This aspect was beyond our control as the tissues were received from brain banks; however, we endeavoured to minimise this interference. In this way, autoradiography studies reflect the in vivo behaviour of a tracer with more accuracy as the tissue’s integrity (along with target accessibility) is preserved. The small frozen brain section autoradiography studies with ^3^H-PI2620, ^3^H-MK6240 and ^3^H-RO948 in FC tissue from CBD and PSP brains explicitly demonstrated this. In both CBD and PSP tissue, there was an evident difference in ^3^H-PI2620 binding intensity between the total and NSP binding sections which was complemented by quantitative analyses, in contrast to previous studies showing no binding in the FC of PSP patients [23]. The specific binding of ^3^H-PI2620 was in the range of 58-77 %, for the three CBD cases, and 44-73 % for the two PSP cases. For CBD and PSP, specific binding values were ~ 3.1- and 2.9-fold higher, respectively, than in brain homogenates (compare Fig. 1 and Table 2; Specific binding). On the adjacent CBD and PSP small frozen brain sections, autoradiography using ^3^H-MK6240 showed a small difference between the total and NSP binding. The specific binding of ^3^H-MK6240 was unequivocally lower than that of ^3^H-PI2620 (~ 17 % in CBD sections and ~ 15 % in PSP sections). For ^3^H-RO948, we observed a difference in intensity between the total and NSP binding; however, the strength of the binding was lower than that for ^3^H-PI2620. Moreover, in small frozen CBD brain sections, ^3^H-PI2620 bound in a diffuse manner, with no frontier between the cortical grey matter and the white matter. Small frozen brain section autoradiography findings were further supplemented by immunostaining studies performed on adjacent sections of CBD and PSP tissue with AT8 (targeting 3R + 4R isoforms). We observed high AT8 immunoreactivity with AT8 in the grey matter (numerous tau threads), glial cells and white matter. AT8 immunoreactivity was higher in the superficial cortical layers of CBD tissue and in the deeper cortical layers of PSP tissue. In a laminar pathology such as AD, neurons are anatomically positioned in different layers in the cortex, which explains the distinctive border between the grey and the white matter observed with the three tracers [58]. In CBD the important glial pathology is more spread explaining why the tracers binding is more diffuse in the grey and white matter. Interestingly, in PSP pathology, even if the glial pathology was highly present, no laminar pattern was observed, and the tracers showed a higher binding in the grey matter compared to white matter (to a lower extent for ^3^H-RO948).

In summary, our findings clearly demonstrate the power of ^3^H-PI2620 to discriminate between AD and control cases, and emphasise that ^3^H-PI2620, ^3^H-MK6240 and ^3^H-RO948 could be valuable for understanding the tau heterogeneity in AD and non-AD tauopathies, through their propensity to target different tau conformations. However, our autoradiography studies may have been limited by the use of only one AD and a few CBD and PSP brains. Further studies in a larger number of cases, including different CBD and PSP subtypes, would be interesting for providing a more mechanistic insight into these primary tauopathies. Moreover, even if we optimised the experimental conditions to the best, we cannot rule out the possibility of variation which can arise from the change of buffer or the process of homogenisation, triggering conformational changes preventing the binding/access of the tracer on tau fibrils. We believe that it is very important to consider several factors and a rigorous standardisation needs to be done while designing these studies. Also, we like to emphasise the need to perform small and large brain section autoradiography studies with binding studies in tandem.

Overall, this study clearly shows that ^3^H-PI2620 has comparable binding affinity in AD, CBD and PSP brain tissue, although the binding site density can vary between these pathologies in the order: AD > CBD > PSP. Our competitive binding studies indicated that PI2620 can detect multiple binding sites in AD, CBD and PSP brain tissue. Moreover, ^3^H-PI2620, ^3^H-MK-6240 and ^3^H-RO948 behave similarly in AD brain tissue, i.e. there are multiple binding sites with equivalent affinities and site proportions as well as similar binding patterns in different brain regions. Most importantly, in CBD and PSP brains, ^3^H-PI2620 displayed high specificity; this was not observed with ^3^H-MK6240. Our results explicitly highlight and support the great complexity of tau proteins (fold/conformation) in different proteinopathies and complement recent biochemical and structural studies demonstrating tau heterogeneity [7, 36]. Additional in vitro and in vivo brain imaging studies focusing on new more selective tau tracers are needed in order to better understand the underlying pathophysiological mechanisms and clinical complexity of AD and non-AD tauopathies.

## Supplementary information

Supplementary Figure

## Data Availability

The raw data used in this study are available from the corresponding authors upon reasonable request.
