# Correction: Discriminative binding of tau PET tracers PI2620, MK6240 and RO948 in Alzheimer’s disease, corticobasal degeneration and progressive supranuclear palsy brains

**DOI:** 10.1038/s41380-023-02021-2

**Published:** 2023-03-08

**Authors:** Mona-Lisa Malarte, Per-Göran Gillberg, Amit Kumar, Nenad Bogdanovic, Laëtitia Lemoine, Agneta Nordberg

**Affiliations:** 1 Division of Clinical Geriatrics, Center for Alzheimer Research, Department of Neurobiology, Care Sciences and Society, Karolinska Institutet, Stockholm, Sweden; 2 Theme Inflammation and Aging, Karolinska University Hospital, Stockholm, Sweden

**Keywords:** Diagnostic markers, Neuroscience

Correction to: *Molecular Psychiatry* 10.1038/s41380-022-01875-2, published online 29 November 2022

After our manuscript has been published online, we identified an error in our semi-quantification values presented in Table 2 of the manuscript, due to an error in the tritium standard reference date. To calculate the amount of tracer bound in fmol/mg, we prepare the linear standard curve using the commercially available tritium standards ranging from 0 to 489.1uCi/g. Since tritium has a half-life of 12.33 years, to know the percentage of activity remaining and respective calculation of fmol/mg, we need to take into consideration the manufacturing date of tritium standards, which was unfortunately mixed up in one of our analysis’s files. Instead of year 2010, we used year 2018 in one file. We have now re-performed the analyses with the correct reference date. The new values are in the same range (see correction in the revised ms and Table 2) and comparable to the published ones. This erratum does not change the final results, interpretation and overall conclusions of the paper but for the sake of accuracy and transparency, we rectified Table 2 and sincerely apologize. The corrected Table 2 is attached.

**Table 2:** Comparative 3H-PI2620 and 3H-MK6240 binding studies expressed as total (fmol/mg), non-specific (NSP; fmol/mg) and specific (fmol/mg and %) binding in small frozen sections from CBD and PSP brains.

Diagnostic group	TOTAL (fmol/mg)		NSP (fmol/mg)		Specific (fmol/mg)		Specific (%)	
	^3^H-PI2620	^3^H-MK6240	^3^H-PI2620	^3^H-MK6240	^3^H-PI2620	^3^H-MK6240	^3^H-PI2620	^3^H-MK6240
CBD 20	47.6	39.1	19.9	29.9	27.6	9.2	58.1	23.6
CBD 22	71.3	44.5	16.5	51.3	54.9	0.0	76.9	0.0
CBD 23	87.6	69.7	25.3	49.8	62.3	19.9	71.1	28.6
PSP 27	47.2	35.6	12.3	29.1	34.9	6.5	73.9	18.4
PSP 28	46.3	39.1	26.0	34.5	20.3	4.6	43.9	11.8

*CBD* cortico basal degeneration, *PSP* progressive supranuclear palsy.

