# Supplementary material for: Discriminative binding of tau PET tracers PI2620, MK6240 and RO948 in Alzheimer’s disease, corticobasal degeneration and progressive supranuclear palsy brains
Source: Mol Psychiatry. 2022 Nov 29;28(3):1272–83. doi: 10.1038/s41380-022-01875-2 (PMC10005967; doi:10.1038/s41380-022-01875-2)
Supplement: Supplementary file 1 — Supplementary Figure [file 41380_2022_1875_MOESM1_ESM.docx]

**Supplementary Fig. 1.**  **Lower magnification of AT8 staining on small frozen post-mortem frontal brain sections from CBD and PSP patients**

AT8 (pS202/pT205) staining (1:1000) performed on adjacent frozen frontal cortex sections from CBD patients 20, 22, and 23, and PSP patients 27 and 28 are presented as pathology references (magnification 2.5 X for all images). Black arrows are pointing out tau present in astrocytic plaques and tufted astrocytes in CBD and PSP brains respectively. The same scale was used for all CBD and PSP samples (500$\mu$m). *CBD = cortical basal degeneration; PSP = progressive supranuclear palsy.*
